# Low blank sampling method for measurement of the nitrogen isotopic composition of atmospheric NO_x_

**DOI:** 10.1371/journal.pone.0298539

**Published:** 2024-02-29

**Authors:** Kazuki Kamezaki, Takahisa Maeda, Shigeyuki Ishidoya, Ayumi Tsukasaki, Shohei Murayama, Naoki Kaneyasu

**Affiliations:** 1 Environmental Management Research Institute, National Institute of Advanced Industrial Science and Technology (EMRI/AIST), Tsukuba, Japan; 2 Fukushima Institute for Research, Education and Innovation, Namie-machi, Fukushima, Japan; University of Saint Andrews, UNITED KINGDOM

## Abstract

The nitrogen isotopic composition of nitrogen oxide (NO_x_) is useful for estimating its sources and sinks. Several methods have been developed to convert atmospheric nitric oxide (NO) and/or nitrogen dioxide (NO_2_) to nitrites and/or nitrates for collection. However, the collection efficiency and blanks are poorly evaluated for many collection methods. Here, we present a method for collecting ambient NO_x_ (NO and NO_2_ simultaneously) with over 90% efficiency collection of NO_x_ and low blank (approximately 0.5 μM) using a 3 wt% hydrogen peroxide (H_2_O_2_) and 0.5 M sodium hydride (NaOH) solution. The 1σ uncertainty of the nitrogen isotopic composition was ± 1.2 ‰. The advantages of this method include its portability, simplicity, and the ability to collect the required amount of sample to analyze the nitrogen isotopic composition of ambient NO_x_ in a short period of time. Using this method, we observed the nitrogen isotopic compositions of NO_x_ at the Tsukuba and Yoyogi sites in Japan. The averaged δ^15^N(NO_x_) value and standard deviation (1σ) in the Yoyogi site was (−2.7 ± 1.8) ‰ and in the Tsukuba site was (−1.7 ± 0.9) ‰ during the sampling period. The main NO_x_ source appears to be the vehicle exhaust in the two sites.

## Introduction

Nitric oxide (NO) and nitrogen dioxide (NO_2_) are collectively referred to as nitrogen oxide (NO_x_). NO_x_ is a primary pollutant in the atmosphere and is involved in urban environmental issues such as photochemical smog, acid rain, tropospheric ozone production, and human health. Besides, NO_x_ deposition can enhance ecosystem productivity through fertilization or decrease it through nutrient imbalances and reduce ecosystem biodiversity through acidification and eutrophication [1]. NO_2_ is oxidized to nitrate (NO_3_^−^), which is adsorbed by aerosols and transported over long distances, affecting distant environments [2]. In areas affected by human pollution, fossil-fuel combustion from traffic, residential heating, cooking, industry, and energy sectors are the main sources of NO_x_. On the other hand, as a natural NO_x_ source, biomass burning, biogenic production, and lightning are also important sources of NO_x_ [3]. Global annual NO_x_ emissions have been gradually curtailed [4]. However, it is important to understand the exact behaviour of NO_x_ to elucidate how the suppression of NO_x_ emissions changes atmospheric reactions.

The nitrogen isotopic composition (δ^15^N value) of NO_x_ is a useful tool for estimating its sources because the nitrogen isotope composition of each source has a unique value (S1 Table). Nitrogen source is identified from the nitrogen isotopic composition of NO_3_^−^ in the aerosol [5–7]. To date, several methods have been developed to convert atmospheric NO and/or NO_2_ to nitrites and/or nitrates for collection. A denuder system, filter pack, and Ogawa sampler have all been used to collect ambient NO_2_ with reagents, such as triethanolamine, guaiacol, and potassium hydroxide [8–13]. A wet method was used to collect NO_x_ by passing air containing NO_x_ through the recovery solution. Potassium permanganate (KMnO_4_) with sodium hydroxide (NaOH) or 20% triethanolamine in water have been used as the recovery solutions for the collection of ambient NO_x_ [14–16]. In addition, a solid sorbent method with attached chemical reagents has also been reported for the collection of ambient NO_x_ [17]. It is important that the collection efficiency of NO_x_ is close to 100% and that the blank is small for the isotopic composition analysis. However, the collection efficiency and blanks are poorly evaluated for many collection methods and the locations at which the δ^15^N values of NO_x_ were measured were limited owing to the difficulty of the measurement method.

Recently, a high-time-resolution method for NO_x_ collection was developed using gas-washed bottles in KMnO_4_ and NaOH recovery solutions. This method shows high collection efficiency for NO_x_, whereas a high concentration of NO_x_ blank (approximately 5 μM) is observed [14, 15]. Therefore, at present, there is almost no fully validated simple method that can collect NO and NO_2_ for the analysis of the δ^15^N values of NO_x_ with high efficiency. Compared to the KMnO_4_/NaOH recovery solution, the hydrogen peroxide (H_2_O_2_)/NaOH recovery solution can remove NO_x_ more efficiently, as reported by Ohta et al. [18] and Kuropka, [19].

In highly alkaline conditions, H_2_O_2_ produces various intermediate products that act as oxidants with H_2_O_2_ decomposition [20]. Free radicals generated due to H_2_O_2_ decomposition efficiently oxidize NO. It has been pointed out that particularly oxygen anions (O_2_^−^) produced at high pH may effectively oxidize NO [21]. On the other hand, NO and NO_2_ dissolve in NaOH solution, and the presence of H_2_O_2_ accelerates the oxidation of NO_2_ [18, 22]. The mechanism of the reaction of NO_x_ with H_2_O_2_/NaOH is expressed as follows:

$$H_{2}O_{2}⇆OOH^{-}+H^{+}$$ (1)

$$H_{2}O_{2}+OH^{-}⇆HOO^{-}+H_{2}O$$ (2)

$$H_{2}O_{2}+HOO^{-}\to OH\cdot +{O_{2}}^{-}\cdot +H_{2}O$$ (3)

$${O_{2}}^{-}\cdot +NO\to ONOO^{-}$$ (4)

$$2NO_{2}+2OH^{-}\to N{O_{2}}^{-}+N{O_{3}}^{-}+H_{2}O$$ (5)

$$N{O_{2}}^{-}+H_{2}O_{2}\to N{O_{3}}^{-}+H_{2}O$$ (6)

In this study, we tested and developed a more efficient NO and NO_2_ collection method using a H_2_O_2_ /NaOH recovery solution for the sampling method and measurement of nitrogen isotopic composition of atmospheric NO_x_. This method has high NO_x_ collection efficiency and low NO_2_^−^ and NO_3_^−^ blanks in the recovery solution.

## Materials and methods

### Commercial NO_x_ samples and recovery solution preparation

Commercial cylinders containing 91 ppm NO (Sample A, Japan Fine Products Co. Ltd., Kanagawa, Japan) and 5 ppm NO_2_ (Sample B, Japan Fine Products Co. Ltd., Kanagawa, Japan) balanced with N_2_ were used in this study. To dilute these high-concentration NO and NO_2_ gases, pure N_2_ (99.995% purity) was used.

To prepare 200 mL of the recovery solution, a highly concentrated 10 M NaOH solution was prepared using reagent-grade NaOH (Special Grade; FUJIFILM Wako Pure Chemical Corp., Osaka, Japan). 10 mL of concentrated NaOH solution was added and diluted with 80 mL of 18.2 MΩ cm water produced by IQ7010 (Merck Millipore Corporation, Massachusetts, United States) in a beaker. Next, 20 mL of 35 wt% H_2_O_2_ (Special grade, FUJIFILM Wako Pure Chemical Corp., Osaka, Japan) was added. The entire solution was made up to 200 mL with an additional 18.2 MΩ·cm water. NaOH was diluted before adding H_2_O_2_, because H_2_O_2_ decomposes rapidly in highly concentrated basic solutions [23]. The old reagents were not used because H_2_O_2_ gradually decomposes; therefore, the prepared reagents were used within one day. In our experimental study, it was observed that refrigerated H_2_O_2_ remained usable for a period of six months following its purchase. However, after a duration of nine months, the H_2_O_2_ failed to generate bubbles even upon NaOH addition, and its NO_x_ trapping efficiency was low. After preparing the reagent, the reaction was allowed to proceed for 30 min to 1 h before the H_2_O_2_/NaOH recovery solution was used (35 wt% H_2_O_2_ was used in this study).

For comparison with the conventional method, we prepared a KMnO_4_ /NaOH recovery solution with reference to Fibiger et al. [14]. Briefly, 1 N (0.2 M) KMnO_4_ was prepared from reagent-grade KMnO_4_ (Special grade, FUJIFILM Wako Pure Chemical Corp., Osaka, Japan). The 125 mL, 1 N KMnO_4_ was diluted to 300 mL, and 25 mL of 10 M NaOH was added. The entire solution was 500 mL with an additional 18.2 MΩ·cm of water. The KMnO_4_/NaOH recovery solution was stored in a 500 mL amber glass bottle and used within one day to prevent contamination with NO_x_.

### Sampling system

**Fig 1:**
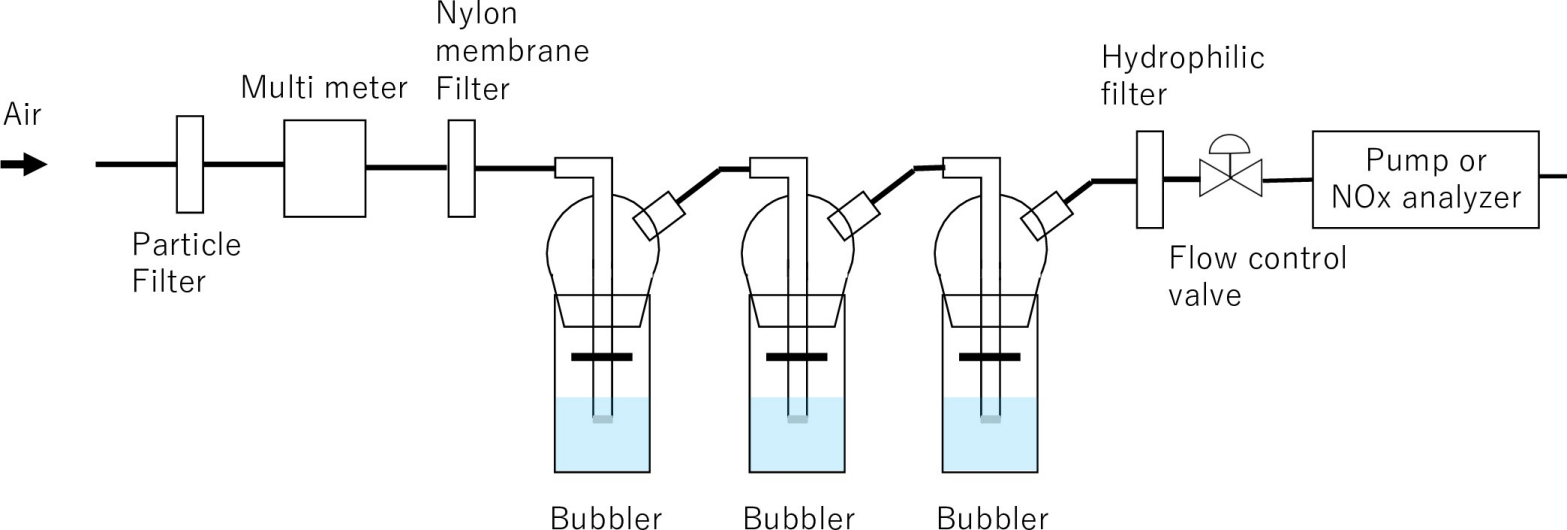
Schematic diagram of the NO_x_ sampling system. When the laboratory experiment was to collect diluted samples A and B, the multi-meter was moved in front of the particle filter.

A schematic of the sampling system is shown in Fig 1. All the tubes were 1/4-inch. Three bubblers (080100–02, SIBATA, Tokyo, Japan) were used to maintain high NO_x_ collection efficiency. A rubber tube was used as a bubbler joint. A PTFE filter (Advantec Co. Ltd., Tokyo, Japan) and a 0.45 μm pore size Whatman’s nylon membrane filter equipped with PFA filter folders (Savillex, Minnesota, United States) were used in front of the bubblers to remove aerosols and gas-phase nitrate (HNO_3_) from the atmosphere. The flow rate was controlled by using a valve immediately prior to the pump. An 8 μm pore size hydrophilic filter (Merck Millipore Ltd., Massachusetts, United States) equipped with a PFA filter folder was placed between the valve and bubblers to prevent water droplets from entering the pump. In addition, it was equipped with a gas flow multi-meter (Model 5210, TSI Incorporated, Minnesota, United States) that can measure pressure, temperature, flow rate, and integrated flow rate, as well as a pump (DAP-12S, ULVAC, Kanagawa, Japan) for atmospheric suction.

### Laboratory experiment

#### NO_x_ collection

To test the NO_x_ collection efficiency, the pump was replaced with a NO_x_ analyser (APNA-370, HORIBA, Kyoto, Japan or Serinus 40, ACOEM ECOTECH, Melbourne, Australia) and a multi-meter was moved in front of the PTFE filter. A three-port valve was installed to bypass the three bubblers. The difference in concentrations between the two instruments was within 1 ppb for NO, NO_2_, and NO_x_ at atmospheric concentrations (< 90 ppb). Cylinders containing NO or NO_2_ were diluted in two steps using pure N_2_ equipped with a mass flow controller (Kofloc, Kyoto, Japan) to prepare approximately 15 and 40 ppb NO_x_ gas. The collection periods were one hour for each sampling and the flow rate was 0.8 L min^−1^. For the sample collection, the 10 mL of recovery solution was placed in three bubblers. For blanks, 5 mL of the solution was placed in three bubblers and collected immediately without sucking air and the blank was collected every 2–3 sample collection. Room temperature was maintained at 22–30°C. Prior to entering the collection system, some of the diluted NO or NO_2_ gas was vented outside to reduce the pressure and adjust the sampling pressure to atmospheric pressure (1000–1020 hPa). After pumping, all recovered solutions were transferred to a 60 mL amber plastic bottle. The collection efficiency was calculated from the reduction in NO and NO_2_ concentrations measured by the NO_x_ analyser with and without passing through the three bubblers.

For the H_2_O_2_/NaOH recovery solution, after transferring the recovery solution to a 60 mL amber plastic bottle, H_2_O_2_ was removed from the recovery solution by adding manganese oxide (MnO_2_) (Special grade, FUJIFILM Wako Pure Chemical Corp., Osaka, Japan) to stop the reaction [24]. It is necessary to slightly loosen the lid of the plastic bottle or periodically open it because oxygen is generated. Hydrogen chloride (HCl) was added to neutralize the recovery solution.

For the KMnO_4_/NaOH recovery solution, we followed the method reported by Fibiger et al. [14]. After transferring the recovery solution to a 60 mL amber plastic bottle for storage, the solution was transferred to a well-washed 500 mL glass beaker. Then, a total of 10 mL of 35 wt% H_2_O_2_ was added to reduce KMnO_4_. When incorporating H_2_O_2_, exercise caution to use a 500 mL glass beaker with a wide mouth instead of the designated 60 mL amber plastic bottle. Failure to do so may result in an abrupt release of the solution. After stopping the reaction, the clear solution and brown MnO_2_ were transferred to a 50 mL centrifuge tube. Then, the solutions were added HCl to neutralize and centrifuged at 4800 rpm for 15 minutes. After centrifugation, the supernatant was collected. MnO_2_ adhering to the beaker can be removed by washing with HCl. Similarly, the sampling system also deposits MnO_2_ and clogs the frit, requiring HCl to be added to remove the MnO_2_ after several samples.

#### Ammonia impact assessment

To test the effect of ammonium ion contamination on the δ^15^N values of NO_x_, 100 μM of NH_4_Cl (special grade, FUJIFILM Wako Pure Chemical Corp., Osaka, Japan) in water was added to the prepared recovery solutions. After seven days, MnO_2_ or H_2_O_2_ was added to the recovery solution to stop the reaction. Continuous flow analysis (CFA) (QuAAtro-2HR, BL TEC K.K., Tokyo, Japan) was used to compare the effect of ammonia mixing by measuring the concentrations of nitrate and nitrite ions in the samples with and without added ammonia.

### Field measurement

Ambient NO_x_ samples were collected at Tsukuba in the west office of AIST (Tsukuba site), Ibaraki, Japan (36.05°N, 140.12°E, 12 m above ground level), and Tokai University in Shibuya (Yoyogi site), Tokyo, Japan (35.66° N, 139.68° E, 52 m above ground level) from January to February 2023 on weekdays (S1 Fig). The Tsukuba site does not require permission as it is the author’s affiliated institution. Access to the Yoyogi site was granted by Tokai University. We confirmed that the field studies did not involve endangered or protected species. The flow rate ranges from 0.5 to 0.8 L min^−1^. After confirming that the NO_x_ concentration did not change, a PFA or Dekabon tube was used to connect the air inlet to the sampling system. It was confirmed at Tsukuba that more than 95% of the NO_x_ had been collected by branching the collection system and measuring the NO_x_ concentrations before and after passing the collection system. The NO_2_^−^ and NO_3_^−^ blanks were about 0.5 μM in the Tsukuba and the Yoyogi sites.

#### Isotopic analysis

Ten nmol of nitrate and/or nitrate ions in the obtained recovery solution were measured using the denitrifier method [25, 26]. If a small amount of MnO_2_ (about 0.2 g) was added stopping the reaction of the H_2_O_2_/NaOH recovery solution, the isotope ratio was not affected even if MnO_2_ dissolved in about 20 mL of solution was directly added to the vial. The automated injection line was modified from Hattori et al. [27] and a schematic diagram of the injection system is depicted in S2 Fig. Briefly, NO_2_^−^ and NO_3_^−^ were converted to nitrous oxide (N_2_O) by a strain of denitrifying bacteria, *Pseudomonas aureofaciens*, which has no N_2_O reductase. The N_2_O produced was then separated from other chemical species using chemical traps and a column equipped with a gas chromatograph (HP-plot Q; Agilent Technologies, Inc., California, United States) and was measured using an isotope-ratio mass spectrometer (IRMS) (MAT252; Thermo Fisher Scientific Inc., Massachusetts, United States) with industrial helium (99.99% purity) as carrier gas. Internationally recognized NO_3_^–^ reference standards USGS 32, 34, 35 and their mixtures were measured alongside the samples and used to correct the resulting mass 45/44 and 46/44 ratios to obtain the final δ^15^N and δ^18^O, respectively. The 1σ analytical uncertainty of δ^15^N and δ^18^O values were ± 0.5 and ± 0.8 ‰, respectively. The low purity of helium affects the measurement precision, but industrial helium was used because ultrapure helium is expensive and not easily available. Although we confirmed with IRMS that the baseline for m/z 44, 45, and 46 did not increase when changing from ultrahigh purity helium to industrial-purity helium, the standard deviation (1σ) of the standard δ^15^N value deteriorated by 0.2‰. Impurities contained in helium were likely concentrated during the N_2_O purge and trap process. Although ultrahigh-purity helium was more suitable for isotope measurement, depending on the molecule, industrial-purity helium was deemed suitable for measurements with little deviation from the blank. Adding MnO_2_ to NO_3_^–^ reference standards did not change the precision and accuracy of δ^15^N values.

#### Definition

Stable isotopic compositions are typically reported as:

$$\delta X_{sample}=\frac{R_{sample}}{R_{standard}}-1,$$ (7)

where *X* denotes ^18^O, and ^15^N, and *R* represents the ratios of ^18^O/^16^O, and ^15^N/^14^N in either the sample or standard material. The δ values are often quoted using per mil (δ) notation. The δ^15^N value was relative to atmospheric N_2_ (air), whereas the δ^18^O value is relative to Vienna Standard Mean Ocean Water (VSMOW). After analysis of the sample recovery solution and blank, the final sample isotopes were calculated using the mass balance:

$$\delta X_{\mathrm{sample}}=(\delta X_{\mathrm{total}}{\left[{{\mathrm{NO}}_{3}}^{-}\right]}_{\mathrm{total}}-\delta X_{\mathrm{blank}}{\left[{{\mathrm{NO}}_{3}}^{-}\right]}_{\mathrm{blank}})/({\left[{{\mathrm{NO}}_{3}}^{-}\right]}_{\mathrm{total}}-{\left[{\mathrm{NO}}_{3}^{-}\right]}_{\mathrm{blank}})$$ (8)

where δ*X*_total_ and δ*X*_blank_ were determined by IRMS with the sample and blank measurement, respectively. [NO_3_^−^] _total_ and [NO_3_^−^] _blank_ were determined by CFA or IRMS with the sample and blank measurement, respectively. For the atmospheric NO_x_ sample, to ensure precise and accurate measurement of the δ^15^N values, we considered the Δ^17^O (Δ^17^O = δ^17^O – 0.52 × δ^18^O) [28] of the analyte N_2_O.

## Results and discussion

### Collection efficiency

Nitrogen isotope exchange between NO and NO_2_ has been suggested to influence N stable isotope compositions. For accurate NO_x_ isotopic composition measurements, nitrogen isotope analysis of atmospheric NO_x_ requires the collection of both NO and NO_2_ with high collection efficiency. Furthermore, because NO and NO_2_ have different physical properties, a differential assessment of NO and NO_2_ collection efficiencies is required. The NO_x_ collection efficiencies are listed in Table 1. After the experiment, like Fibiger et al. [14], the volume of the solution decreased by a few mL, indicating droplet dispersal. To prevent loss of nitrate due to droplet scattering, three bubblers were used, although the collection rate did not differ considerably when two bubblers were used. Although the effect of this decrease in water content on the isotopic composition of nitrate is difficult to estimate, the concentration of nitrate in the third bubbler was the same as that in the blank. Droplet dispersal is mainly affected by the third-stage bubbler, but since the third stage has a low NO_x_ concentration, the effect of droplet dispersal on the isotopic composition of nitrate was deemed to be negligible.

**Table 1:** Collection efficiency of NO and NO_2_ by H_2_O_2_/ NaOH and KMnO_4_/NaOH recovery solution.

	Recovery solution (10 mL solution in three bubblers)	
Gas	H_2_O_2_/NaOH^a^	KMnO_4_/NaOH^b^
***15–20 ppb NO***_***x***_		
**NO**	97 ± 4% (*n* = 5)	85 ± 3% (*n* = 4)
**NO**_**2**_	99 ± 3% (*n* = 5)	91 ± 3% (*n* = 4)
***30–40 ppb NO***_***x***_		
**NO**	94 ± 2% (*n* = 8)	83 ± 4% (*n* = 5)
**NO**_**2**_	97 ± 3% (*n* = 8)	91 ± 2% (*n* = 5)

^a^H_2_O_2_/NaOH recovery solution was prepared as a mixture of 3 wt% H_2_O_2_ and 0.5 M NaOH. ^b^KMnO_4_/NaOH was prepared by mixtures of 0.25 M KMnO_4_ and 0.5 M NaOH. The average and standard deviation (1σ) of collection efficiency was calculated from the reduction in NO and NO_2_ concentrations measured by the NO_x_ analyzer with and without passing through the three gas bubblers.

No significant difference was found when comparing the collection efficiency of both recovery solutions at NO_x_ concentrations of 15 and 40 ppb (Table 1). The overall averaged H_2_O_2_/NaOH recovery solution collected over 90% of NO and over 95% of NO_2_. Over 90% of NO and NO_2_ collection efficiency using H_2_O_2_/NaOH recovery solution was also reported by Ohta et al. [18], when the concentration of the recovery solution was over 0.6% H_2_O_2_ and 0.24 M NaOH. Note that while air is flowing, CO_2_ reacts with NaOH in solution, lowing the pH [21, 22]. Thus, the concentration of NaOH should be greater than 0.24 M. More than 95% of NO_x_ can be collected by using H_2_O_2_/NaOH recovery solution since NO_2_/NO_x_ in the atmosphere mostly exceeds 50%. The high collection efficiency of the H_2_O_2_/NaOH recovery solution probably made isotopic fractionation negligible during sampling. However, it is necessary to consider the differences in isotopic composition due to the differences in the collection efficiencies of NO and NO_2_. The reported δ^15^N(NO_2_) values ranges from −22 to 5‰ [10–12, 17]. Since fractionation factors for ^15^N substitution between NO and NO_2_ ranged from 1.040 at 278 K to 1.034 at 310 K [29], when the δ^15^N(NO_2_) values are from −22 to 0.4 ‰, the expected δ^15^N(NO) values is higher by approximately 40‰ at 278 K. The apparent δ^15^N(NO_x_) value can be increased by a maximum of 0.74 ‰ compared to the true value by the difference in the collection efficiency of NO and NO_2_ when the mole fraction of NO_2_ to NO_x_ is 0.49.

The KMnO_4_/NaOH recovery solution captured approximately 83–85% of NO and 91% of NO_2_. Given that the mole fraction of NO_2_ is much larger than NO, the collection efficiency of NO_x_ in KMnO_4_/NaOH recovery solution was in good agreement with the reported value (92 ± 10) % [14, 15]. In this study, both recovery solutions can collect NO_x_ with high efficiency, and H_2_O_2_/NaOH has a higher absorption efficiency than KMnO_4_/NaOH under the same conditions. However, we have not evaluated the extent to which recovery solution affects isotope ratio fractionation.

### Nitrite and nitrate concentration in the recovery solution

Blank reduction is important in NO_x_ isotope measurements. We compared the NO_2_^−^ and NO_3_^−^ blanks in H_2_O_2_/NaOH with those in the KMnO_4_/NaOH recovery solution. The NO_2_^−^ and NO_3_^−^ concentrations of the blanks were measured using CFA, and the results are listed in Table 2. The blanks of the H_2_O_2_/NaOH and KMnO_4_/NaOH recovery solution were approximately 0.5 μM and 2.8 μM, respectively. The blank concentrations of NO_2_^−^ and NO_3_^−^ in the H_2_O_2_/NaOH recovery solution were clearly lower than the values of KMnO_4_/NaOH, indicating that the H_2_O_2_/NaOH recovery solution is superior in blank suppression. Fibiger et al. [14] also tried NO_x_ collection using an H_2_O_2_/NaOH recovery solution and found a high nitrate blank (approximately 25 μM). However, such high-level blanks in the H_2_O_2_/NaOH recovery solution were not observed when using the reagents or experimental scheme presented in this study.

**Table 2:** Nitrite and nitrate concentrations of the blank and seven days later after adding ammonium ion in recovery solution.

Recovery solutions		NO_2_^−^ + NO_3_^−^ (μM)^a^	Averaged NO_2_^−^ + NO_3_^−^ (μM)
**Blank**			
	H_2_O_2_/NaOH^b^_1	0.4	0.5 ± 0.2
	H_2_O_2_/NaOH_2	0.8	
	H_2_O_2_/NaOH_3	0.4	
	H_2_O_2_/NaOH_4	0.2	
	H_2_O_2_/NaOH_5	0.5	
	H_2_O_2_/NaOH_6	0.5	
	H_2_O_2_/NaOH_7	0.5	
	KMnO_4_/NaOH_1	1.1	1.5 ± 0.6
	KMnO_4_/NaOH_2	1.0	
	KMnO_4_/NaOH_3	1.7	
	KMnO_4_/NaOH_4	1.4	
	KMnO_4_/NaOH_5	2.3	
**After adding 100 μM ammonium ion** ^**d**^			
	H_2_O_2_/NaOH_1	0.7	0.5 ± 0.2
	H_2_O_2_/NaOH_2	0.6	
	H_2_O_2_/NaOH_3	0.4	
	H_2_O_2_/NaOH_4	0.6	
	H_2_O_2_/NaOH_5	0.3	
	H_2_O_2_/NaOH_6	0.4	
	H_2_O_2_/NaOH_7	0.4	
	KMnO_4_/NaOH_1	3.2	2.8 ± 0.9
	KMnO_4_/NaOH_2	2.4	
	KMnO_4_/NaOH_3	2.1	
	KMnO_4_/NaOH_4	2.7	
	KMnO_4_/NaOH_5	1.9	

^a^The NO_2_^−^ and NO_3_^−^ concentrations in recovery solution were measured by the CFA method. ^b^H_2_O_2_/NaOH recovery solution was a prepared mixture of 3 wt% H_2_O_2_ and 0.5 M NaOH. ^c^KMnO_4_/NaOH recovery solution was a prepared mixture of 0.25 M KMnO_4_ and 0.5 M NaOH.

^d^The reaction was stopped seven days after the addition of ammonium ions, and NO_2_^−^ and NO_3_^−^ concentrations in the recovery solution were measured.

In addition, 120 μM NH_4_Cl was added to KMnO_4_/NaOH and H_2_O_2_/NaOH recovery solutions and allowed to stand for one week to investigate the effect of ammonium ions. Table 2 presents the results of the study. Total NO_2_^−^ and NO_3_^−^ concentration increased in the KMnO_4_/NaOH recovery solution but not in the H_2_O_2_/NaOH recovery solution. As described by Fibiger et al. [14] and Wojtal et al. [15], the KMnO_4_/NaOH recovery solution slightly reacted with ammonia ions after seven days, but the H_2_O_2_/NaOH recovery solution did not react with ammonia ions. However, since neither of the recovery solutions reacted by even 1% of the amount of ammonia added, it is hypothesized that a negligible reaction occurred with ammonia. From these results, it is evident that the H_2_O_2_/NaOH recovery solution is superior to the KMnO_4_/NaOH recovery solution in terms of NO_x_ collection. Subsequent experiments were performed using the H_2_O_2_/NaOH recovery solution only and had a high collection rate and suppressed blanks. Under the same experimental conditions, the H_2_O_2_/NaOH recovery solution outperforms the KMnO_4_/NaOH recovery solution. A further advantage over previous methods is that neutralization can be performed in one vessel, and no centrifugation is required, resulting in a reduced risk of sample loss and contamination.

### Nitrogen isotope measurement for cylinder NO_x_

The δ^15^N values of samples A and B recovered with the H_2_O_2_/NaOH solution were measured, as shown in Table 3. The repeatability of δ^15^N values for samples A and B were (0.7 ± 0.5) ‰ (*n* = 5) and (−18.0 ± 0.8) ‰ (*n* = 5) for 15–20 ppb of NO_x_, respectively. The repeatability of δ^15^N values for samples A and B were (0.8 ± 0.5) ‰ (*n* = 8) and (−17.7 ± 0.7) ‰ (*n* = 8) for 30–40 ppb of NO_x_, respectively. The larger uncertainty of the δ^15^N values for sample B compared to those of sample A is thought to be due to slight contamination from ambient air.

**Table 3:** Reproducibility of δ^15^N value for NO and NO_2_ in the cylinders using H_2_O_2_/NaOH recovery solution.

	Number of experiments	NO_x_ cylinder	Averaged [NO_x_](ppb)	δ^15^N (‰)	Averaged Blank/ total N^c^
**15–20 ppb NO**	**5**	**A** ^**a**^	**NO**_**x**_ **:15**	0.7 ± 0.5	**0.13**
			**(NO:14, NO**_**2**_ **:0)**		
**30–40 ppb NO**	**8**	**A**	**NO**_**x**_ **:32**	0.8 ± 0.5	**0.12**
			**(NO:31, NO**_**2**_ **:1)**		
**15–20 ppb NO**_**2**_	**5**	**B** ^**b**^	**NO**_**x**_ **:16**	−18.0 ± 0.8	**0.06**
			**(NO:2, NO**_**2**_ **:14)**		
**30–40 ppb NO**_**2**_	**8**	**B**	**NO**_**x**_ **:37**	−17.7±0.7	**0.09**
			**(NO:7, NO**_**2**_ **:30)**		

^a^Sample A was 91 ppm NO balanced with N_2_ in a cylinder.

^b^Sample B was 5 ppm NO_2_ balanced with N_2_ in a cylinder. These high concentrations of NO or NO_2_ gases were diluted by pure N_2_ and the diluted concentration was approximately 40 ppb for NO_x_. The collection periods were one hour for each sampling and flow rates were 0.8 L min^−1^.

^c^The blank/total N values were calculated from the sample and blank peak areas using MAT252.

### Quantification of the influence of Δ^17^O on δ^15^N values

The δ^15^N value of N_2_O measured by IRMS is calculated assuming Δ^17^O (= δ^17^O – 0.52 δ^18^O) is 0‰. However, since the Δ^17^O value of N_2_O may apparently increase the δ^15^N value, a correction was performed taking the Δ^17^O value into account. In this study, the Δ^17^O value of NO_3_^−^ was not measured. On the other hand, the maximum δ^18^O value of N_2_O converted from NO_x_ was 40‰ (containing laboratory and field experiments). Note that the δ^18^O value of N_2_O does not directly reflect the δ^18^O value of NO_x_ because NO_x_ obtains oxygen derived from water or H_2_O_2_ during oxidation in the recovery solution. The Δ^17^O values are generated only by mass transfer of O atoms from ozone to products during oxidation reactions [30]. In Eqs 1–6, the Δ^17^O of O supplied during the process of NO and NO_2_ oxidizing NO_3_ is 0‰. Considering that NO and NO_2_ each receive two oxygen atoms, the Δ^17^O values become 2/3 or less (Eqs 1–6). Further, it is assumed that the relationship between the Δ^17^O and δ^18^O values of NO_2_ is 0.36:1 (estimated by a straight line passing through the origin based on the values reported by Albertin et al. [12]). At this time, the maximum Δ^17^O value of N_2_O was 9.5‰ when the δ^18^O value was 40‰. The effect of this Δ^17^O value on the δ^15^N value was estimated using USGS34 and USGS35 with known Δ^17^O values. Based on the result of USGS35 measurements, when the Δ^17^O value is 21.56‰ [28], the apparent δ^15^N value would increase by 1.2‰. This result showed good agreement with the description by Yu and Elliott [16]. Assuming a linear relationship between Δ^17^O and δ^15^N values of USGS34 (Δ^17^O value: −0.3‰ [31]) and USGS35, the Δ^17^O value of 9.5‰ will increase the δ^15^N value by 0.5‰ at maximum. The overall measured 1σ uncertainty of δ^15^N(NO_x_) was ± 1.2‰ by combining the difference in the absorption efficiency of NO and NO_2_, the repeatability, and the consideration of Δ^17^O value.

### Limitations of NO_x_ collection

We also tested the limitations of this developed method. Possible factors that reduce the yield of NO_x_ include a decrease in oxidant concentration due to reaction with NO_x_ and other gases, and a decrease in pH due to reaction with CO_2_. The reaction between NO_x_ and the oxidant agent is not rate-limiting as the input H_2_O_2_ concentration is sufficiently high compared to the NO_x_ concentration. In fact, we tried flowing 40 ppb of NO and NO_2_ for over 12 h each, but the collection efficiency did not fall below 90%. On the other hand, when the time required for the collection rate to drop below 90% was measured for continuous collection of approximately 15 ppb of NO_x_ in air at an average of 0.6 L min^−1^, the collection rate dropped sharply at 14 h (S3 Fig). CO_2_ dissolves in the form of CO_3_^2-^ at high pH as follows:

$$CO_{2}+2OH^{-}\to C{O_{3}}^{2-}+H_{2}O.$$ (9)

Since it is difficult to calculate the dissolution rate of CO_2_ in this study, we assumed that all CO_2_ dissolves in solution. In addition, although OH^−^ ions are used or provided in the decomposition of H_2_O_2_, this was not accounted for. We set the flow rate at 0.6 L min^−1^ and the CO_2_ concentration at 400 ppm. Given that the NO collection efficiency decreases under pH 11 [21] and that the two containers were sufficient to collect NO_x_, the combined NaOH from the two containers would be neutralized in about 8 h. The actual capacity was longer than 8 h, as it is unknown whether all the CO_2_ will be absorbed and whether the first bubbler can continue to collect CO_2_ even after neutralization. When collecting NO_x_ from air, we recommend up to 8 and 6 h for the collection time, at a flow rate of 0.6 and 0.8 L min^-1^, respectively. If used in an environment with high CO_2_ concentration, the flow rate must be reduced, or the number of bubblers must be increased.

### Comparison with the previous NO_x_ isotope measurement method

The advantage of this method is that both NO and NO_2_ can be collected with a low blank of NO_3_^−^; thus, the δ^15^N values of atmospheric NO_x_ can be directly estimated compared to the methods that can only collect NO_2_. An offline method for converting high concentrations (over 100 ppm) of NO_x_ to NO_3_^−^ using H_2_O_2_/NaOH recovery solution was used by Heaton [32]. However, the concentration of H_2_O_2_ and NaOH in the recovery solution is unknown. This study is the first to investigate the NO_x_ collection efficiency, the degree of blanking, and the influence of ammonium ions being quantified using H_2_O_2_/NaOH recovery solution. Another offline wet method for collecting high concentrations of NO_x_ is the use of H_2_SO_4_/H_2_O_2_. However, Chin et al. [33] showed that the 6 wt% H_2_O_2_ in low pH (2 to 4) converted less than 5% of NO in the flue gas, and Ohta et al. [18] showed that the NO_2_ collection efficiency using H_2_O_2_/NaOH recovery solution can be degraded at NaOH concentrations below 0.24 M, suggesting that probably the H_2_O_2_ in basic solution is necessary for high collection efficiency of NO_x_ online. Further advantages of this method are its portability, simplicity, and the ability to collect the required amount of sample to analyze the nitrogen isotopic composition of ambient NO_x_ in a short period of time.

### Nitrogen isotope measurement for atmospheric NO_x_

**Fig 2:**
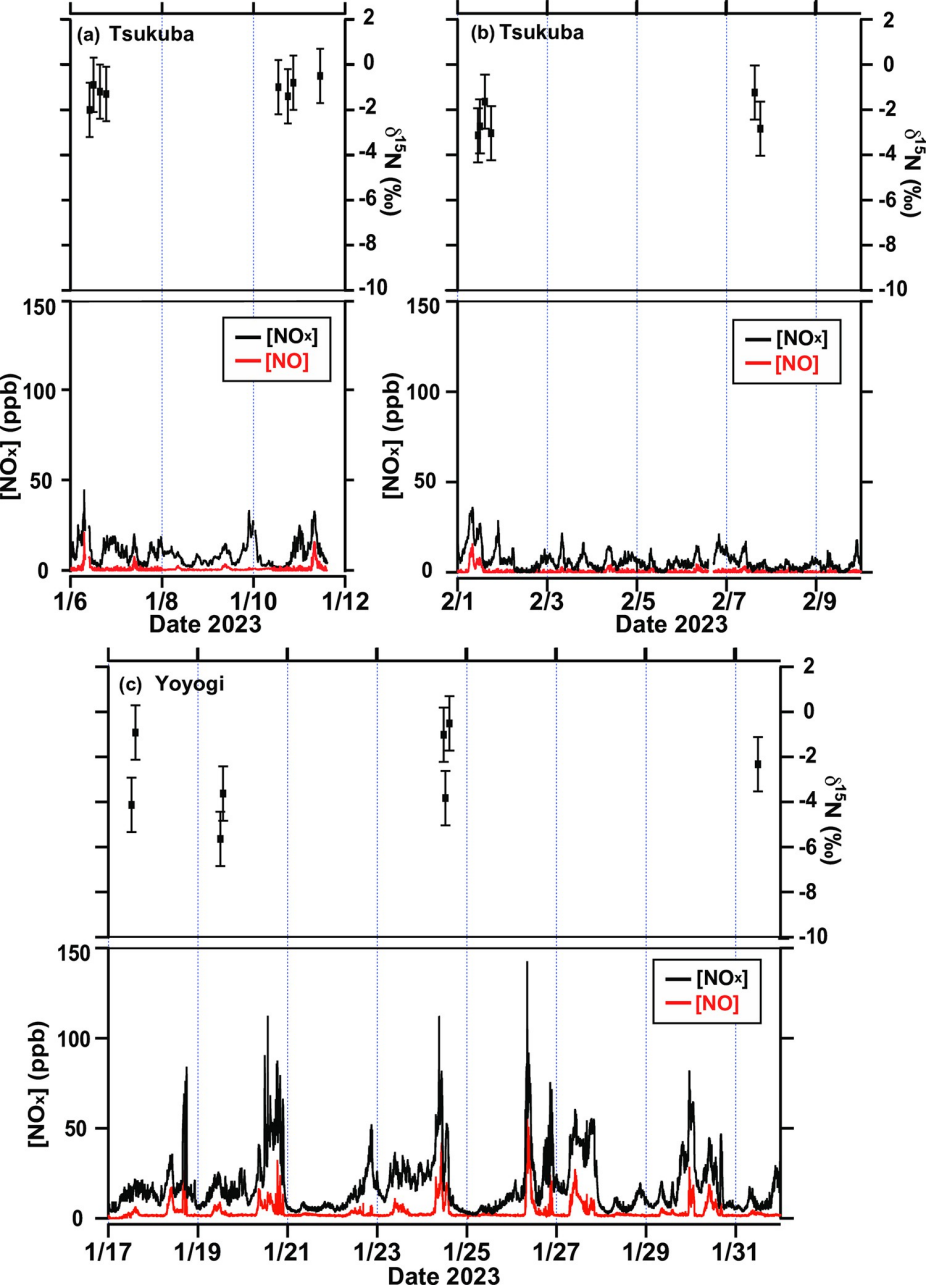
NO (red) and NO_x_ (black) concentrations and δ^15^N(NO_x_) values at the Tsukuba (a, b) and Yoyogi site (c). Error bars indicate 1σ uncertainty of δ^15^N(NO_x_) ± 1.2‰.

The observed δ^15^N values and NO_x_ concentrations for atmospheric NO_x_ collected at the Tsukuba and Yoyogi sites are shown in Fig 2 and S2 Table. The average NO_x_ concentrations during the sampling period were 6 and 18 ppb at Tsukuba and Yoyogi, respectively. The maximum NO_x_ concentrations at Tsukuba and Yoyogi were 45 and 143 ppb, respectively. The diurnal variation in the NO_x_ concentration on weekdays during the sampling period clearly showed two peaks corresponding to traffic rush hours (S4 and S5 Figs).

The δ^15^N(NO_x_) value ranged from −3.1 to −0.5 ‰ at the Tsukuba site and from −5.6 to −0.5 ‰ at the Yoyogi site. The averaged δ^15^N(NO_x_) value and standard deviation (1σ) in the Yoyogi site was (−2.7 ± 1.8) ‰ and in the Tsukuba site was (−1.7 ± 0.9) ‰ during the sampling period, and no significant difference between the two sites was observed. No significant correlation was found between the NO_x_ concentrations or 1/[NO_x_] and δ^15^N(NO_x_) values. Additionally, it also did not correlate with the ratio of NO_2_ to NO_x_ observable when only the δ^15^N(NO_2_) was measured because we collected both NO and NO_2_.

Walters et al. [34] showed the mass-weighted δ^15^N(NO_x_) values emitted from vehicles have the following relationship with vehicle runtime:

$$\delta^{15}N(NO_{x})=-12.35+3.02ln(t+0.455)$$ (10)

where δ^15^N(NO_x_) represents the mass-weighted δ^15^N(NO_x_) values emitted from vehicles and *t* is the vehicle run time (min). Because the average distance of one car in Japan is approximately 20 km (Ministry of Land, Infrastructure, Transport and Tourism website, https://www.e-stat.go.jp/stat-search/files?stat_infid=000032211818, The first summary table by fuel/vehicle type last access: 28 March 2023 [35]), the driving time is expected to be 10−30 mins each. The predicted mass-weighted δ^15^N(NO_x_) values emitted from vehicles (−5.3 to −2.0 ‰; S1 Table) based on Eq 10 were matched with the δ^15^N(NO_x_) values in the Tsukuba and the Yoyogi sites. Therefore, if the main source of NO_x_ is vehicle exhaust, then the δ^15^N(NO_x_) values and diurnal variations in NO_x_ concentrations can be explained. Biomass burning (−7 to 12 ‰ [36]; S1 Table) is also a candidate for the NO_x_ sources of observed δ^15^N(NO_x_) values in the Tsukuba and the Yoyogi sites. However, because the NO_x_ emitted from biomass burning is temporary, it is unlikely to be the main source of NO_x_ in urban areas. Additionally, biomass burning did not show diurnal variation in NO_x_ concentrations, as shown in S4 and S5 Figs. This indicates that the main NO_x_ source collected at the Tsukuba and Yoyogi sites was vehicle exhaust during the sampling period. However, the sampling period is limited and not all variations in δ^15^N(NO_x_) value can be explained. Future investigations are needed to understand NO_x_ dynamics by measuring the δ^15^N(NO_x_) value of NO_x_ sources to enrich the database in the surrounding environment and through long-term observations with higher time resolution.

## Conclusion

We developed a portable new method to collect NO_x_ for nitrogen isotopic measurement by mixing 3 wt% H_2_O_2_ and 0.5 M NaOH solution with a precision (1σ uncertainty) of ± 1.2 ‰. The method using the developed H_2_O_2_/NaOH recovery solution has high NO_x_ collection efficiency, a relatively simpler measurement procedure, small blanks, and a negligible impact of ammonium contamination.

The δ^15^N(NO_x_) values were observed in two sites in Japan. The averaged δ^15^N(NO_x_) value and standard deviation (1σ) in the Yoyogi site was (−2.7 ± 1.8) ‰ and in the Tsukuba site was (−1.7 ± 0.9) ‰ during the sampling period. The main NO_x_ source appears to be the vehicle exhaust in the two sites. However, the sampling period is limited and the not all variations in δ^15^N(NO_x_) value can be explained. Future investigations are needed to understand NO_x_ dynamics by measuring the δ^15^N(NO_x_) value of NO_x_ sources to enrich the database in the surrounding environment and through long-term observations with higher time resolution. In addition, by combining the concentration and δ^15^N value of ammonia, organic nitrogen, and nitrate in aerosols, among others, the understanding of the nitrogen cycle, including NO_x_ will be deepened.

## Supporting information

S1 Fig Aerial view of the sampling sites. (PDF)

S2 Fig Schematic diagram of the system for measuring nitrogen isotope ratios within N_2_O. (PDF)

S3 Fig Limitation of NO_x_ collection. A NO_x_ analyzer was connected behind the bubbler and when approximately 15 ppb of NO_x_ in the air was continuously captured at an average rate of 0.6 L min^-1^, the time required for the collection efficiency to drop below 90% was measured. We set the bubbler at time 0 min.
(PDF)

S4 Fig Box-and-whisker plots of diurnal variation of NO_x_ concentrations during sampling periods (1/4-6, 10–11, 31, 2/1-3, 7–10) at the Tsukuba site. (PDF)

S5 Fig Box-and-whisker plots of diurnal variation of NO_x_ concentrations during sampling periods (1/17-20, 24–27, 30–31) at the Yoyogi site. (PDF)

S1 Table Overview of nitrogen isotopic composition (δ^15^N) for nitrogen oxides (NO_x_) in the atmosphere. (XLSX)

S2 Table The δ^15^N(NO_x_) and NO_x_ concentration of samples collected in ambient urban air at Tsukuba and Yoyogi site from January to February 2023. (XLSX)
