# Supplementary figures and images for: Low blank sampling method for measurement of the nitrogen isotopic composition of atmospheric NOx
Source: PLoS One. 2024 Feb 29;19(2):e0298539. doi: 10.1371/journal.pone.0298539 (PMC10903869; doi:10.1371/journal.pone.0298539)

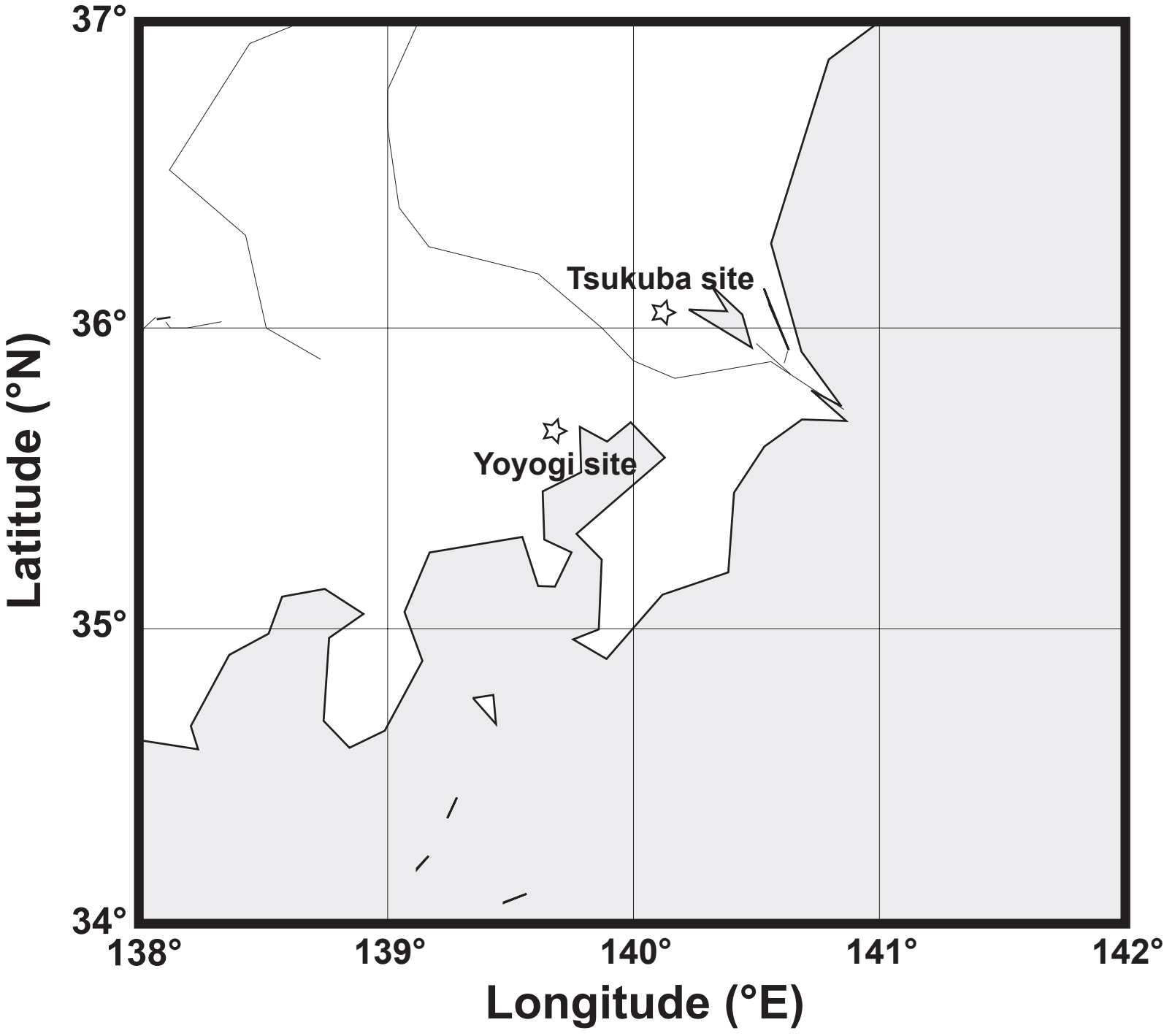

Supplement: S1 Fig — (PDF) [file pone.0298539.s001.pdf]

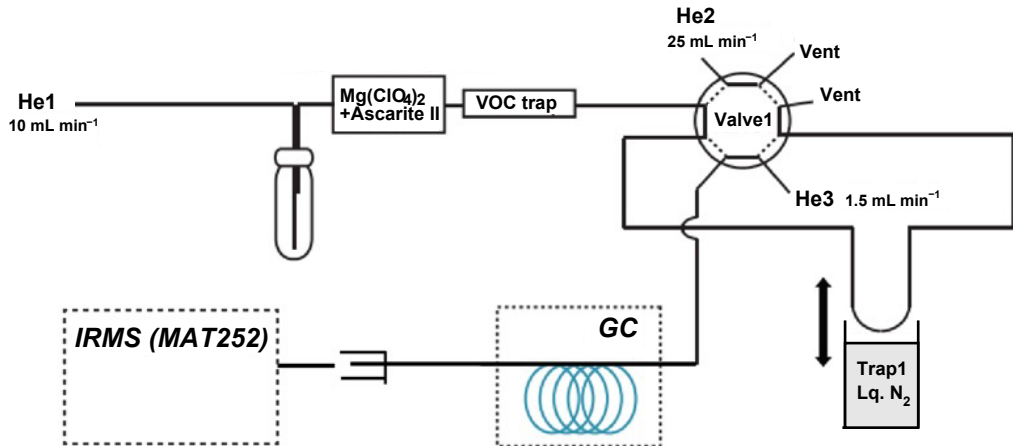

Supplement: S2 Fig — (PDF) [file pone.0298539.s002.pdf]

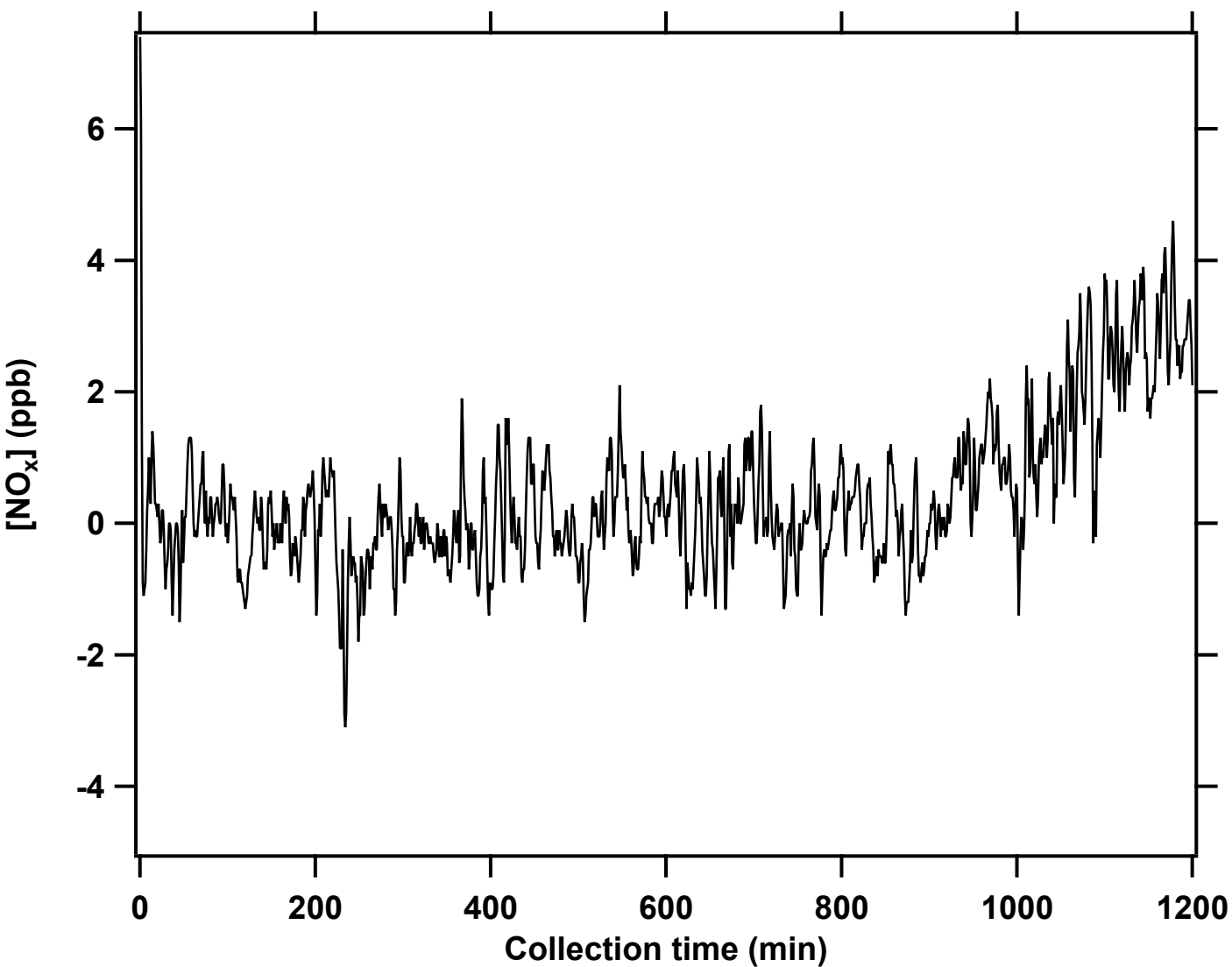

Supplement: S3 Fig — A NOx analyzer was connected behind the bubbler and when approximately 15 ppb of NOx in the air was continuously captured at an average rate of 0.6 L min-1, the time required for the collection efficiency to drop below 90% was measured. We set the bubbler at time 0 min. (PDF) [file pone.0298539.s003.pdf]

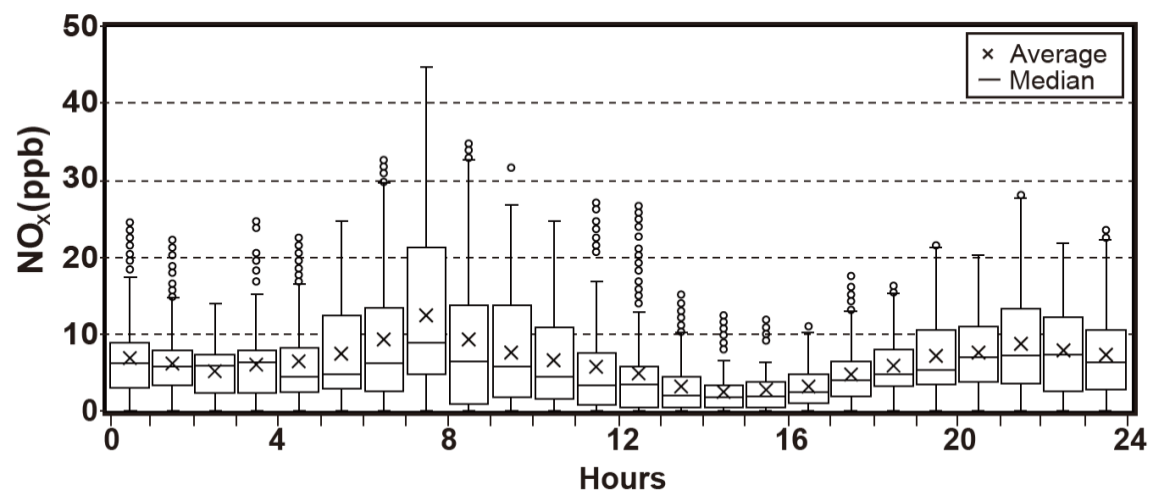

Supplement: S4 Fig — (PDF) [file pone.0298539.s004.pdf]

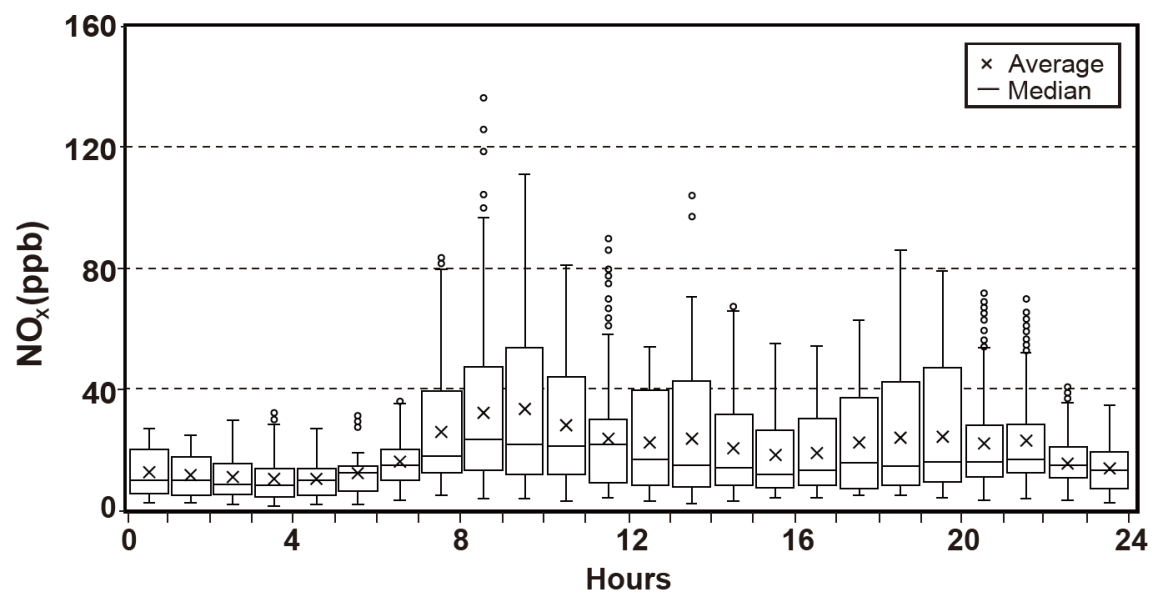

Supplement: S5 Fig — (PDF) [file pone.0298539.s005.pdf]
